# Lattice complex assembled by noncompetitive anti-EGFR antibodies regulates actin cytoskeletal reorganization

**DOI:** 10.1186/s12935-020-01204-z

**Published:** 2020-04-21

**Authors:** Dianshuai Huang, Tianqi Lu, Xingyu Du, Xi Xi, Xin Zhang, Xitian Zhang, Haoran Zhang, Fei Sun

**Affiliations:** 1 Institute of Frontier Medical Science, Jilin University, No.1163 Xinmin Street, Changchun, 130021 Jilin People’s Republic of China; 2 Department of Neurosurgery, Gaoyou Hospital Affiliated Soochow University, Gaoyou People’s Hospital, No.116 Fuqian Street, Gaoyou, 225600 Jiangsu People’s Republic of China; 3 Changchun Intellicrown Pharmaceutical Co., Ltd, No.1688 Jichang Road, Changchun, 130507 Jilin People’s Republic of China

**Keywords:** EGFR, Lattice complex, Noncompetitive antibody combination, Cytoskeleton, PIP2

## Abstract

**Background:**

Recent evidence of clinical trials highlights that the combination of two noncompetitive anti-EGFR antibodies can benefit patients with several cancers. Previous studies propose that a lattice complex assembled by antibodies and EGFR down-regulates surface EGFR by rapid internalization of the complex. However, there remains a paucity of evidence and understanding on the existence of a lattice complex on cell surface and its cellular processes of internalization.

**Methods:**

Herein, we used three dimensions structured illumination microscopy to directly observe the actual morphology of the lattice complex formed on Hela cell membrane after noncompetitive anti-EGFR antibody combinations, and we explored the internalized mechanism of noncompetitive antibody combinations by constructing a PIP2 consumption system.

**Result:**

We observed the lattice complex (length > 1 μm) on the surface of living cell after preincubation with Cetuximab and H11, but combination of Cetuximab and single domain antibody 7D12 fails to assemble the lattice, these results demonstrates the importance of symmetrical structure of conventional antibody for lattice formation. Interestingly, the lattice complex assembles along with cytoskeletal fibers, and its internalization recruits a large amount of PIP2 and triggers the rearrangement of F-actin.

**Conclusions:**

The above data suggests that large-size lattice complex affects membrane fluidity and dynamic reorganization of cytoskeletal, which may be responsible for its rapid internalization. These new insight will aid in current rational combination design of anti-EGFR antibodies.

## Background

In recent years some reports addressed the possibility that anti-EGFR mAb combinations better inhibit tumor growth because they effectively down-regulate receptors on the cell surface [1–4]. A synergistic antibody combination containing two recombinant mAbs that bind to different epitopes of EGFR, was called as Sym004 [5, 6]. In 2017, the data from randomized Phase II of Sym004 in patients with late-stage metastatic colorectal cancer (mCRC) were reported that have acquired resistance to anti-EGFR antibody therapies [7–9]. It can be seen that rapid internalization and recycling blockage of EGFR induced by antibody combination may be a novel strategy to develop anti-EGFR agents.

Wittrup and Yarden et al. reported that the combination of two anti-EGFR monoclonal antibodies with noncompetitive epitopes could induce amount internalization of EGFR, and block the intracellular recycling, which is distinct from ligand-induced endocytosis [10, 11]. Based on the symmetrical structure and two variable regions of antibody, they proposed that these two antibodies conjugate EGFR on cell membrane to form a lattice-like complex. So far, very little attention has been paid to these issues, the existence of lattice complex, the relation between the complex and the rapid internalization, the change of membrane and cytoskeleton in the process of internalization.

This study aims to contribute to this growing area of research by exploring the binding and subsequent behavior of EGFR and antibodies. By 3D-SIM imaging, we showed that combination of noncompetitive antibodies could conjugate EGFR to form large lattice complex. Interestingly, it is assembled along actin, we propose that the large size lattice complex affect actin cycle, so a PIP2 consumption system is used to investigated actin cycle and cell membrane transport. The assembling of noncompetitive antibodies and EGFR recruit a large amount of PIP2, mediate transport of cell membrane and F-actin polymerization, then increase endocytosis of EGFR by high-intensity macropinocytosis. These findings contribute in several ways to our understanding of rapid internalization of EGFR and provide a theoretical basis for the selection and therapeutic regimen of antibody combination.

## Materials and methods

### Cell culture

The Hela cells (ATCC^®^ CCL-2™) and CaSki cells (ATCC^®^ CRM-CRL-1550™) were purchased from the American Type Culture Collection (Manassas, VA, USA), and maintained in Dulbecco’s modified eagle medium (DMEM; Gibco, Thermo Fisher, Waltham, MA, USA), supplemented with 10% heat-inactivated fetal bovine serum (FBS; Gibco), 100 µg/ml streptomycin and 100 U/ml penicillin (Sigma-Aldrich, St. Louis, MO, USA) on 10 cm plates at 37 °C in presence of 5% CO_2_.

### Reagents and chemicals

H11 antibody (Cat No.MA1-12693), 111.6 antibody (Cat No.MA5-13269), Lipofectamine 2000 (Cat No.11668019), Opti-MEM (Cat No.31985088), AlexaFluor 488 Phalloidin (Cat No.A12379) were from Thermo Fisher (Waltham, MA, USA). Cetuximab (Cat No.TM-Cetu-00002) was from Merck (Kenilworth, NJ, USA). Hoechst 33342 (Cat No.B2261), Chlorpromazine (Cat No.C0982), Nystatin (Cat No.N9150), Progesterone (Cat No.P0130) and Rapamycin (Cat No.V900930) were purchased from Sigma-Aldrich. EIPA (Cat No.sc-202458) was from Santa Cruz Biotechnology (Dallas, Texas, USA).

### Selection of competitive and noncompetitive antibody combinations

Based on the previous report on binding sites of anti-EGFR mAbs [11], the binding sites of Cetuximab, H11 and 111.6 were all located on EGFR extracellular Domain III. Cetuximab mapped on residues of Q384, Q408, H409, K443, K465, I467 and N473. H11 mapped on S356 and H359, with 111.6 on R353, S356 and H359. Thus Cetuximab + H11 and Cetuximab + 111.6 were chose to be noncompetitive antibody combination, H11 + 111.6 was used as competitive antibody combination.

### Super-resolution three-dimensional Structured Illumination Microscopy imaging and analysis

Cells grown on glass coverslips were serum-starved for 4 h or not. Then, single antibody or combination of antibodies were added for various times. Next, cells were fixed with 4% paraformaldehyde in PBS for 20 min. And the cells were permeabilized with 0.1% triton for 10 min at room temperature selectively based on the observation of lattice complex or internalization, followed by incubation with related secondary fluorescent antibodies.

Cells were imaged using a DeltaVision OMX Imaging System with the 3D-SIM model (GE Healthcare, No.OM06051, Pittsburgh, PA, USA). Channel, mode, exposure, excitation, and luminousness were set for optimum imaging. SoftWoRx (GE Healthcare) was used to reconstruct image data from OMX. Volume rendering of reconstructive image was also obtained by using softWoRx. Imaging rendering and analysis was carried out using the Imaris software from Bitplane (Zurich, Switzerland).

### Chemical inhibitors

Cells were incubated on 20 mm dishes. Before addition of antibodies, cells were pre-treated with different inhibitors for 30 min. The inhibitor concentrations were as follows: EIPA 100 μM (an inhibitor of macropinocytosis), Chlorpromazine 25 μM (an inhibitor of clathrin-dependent pinocytosis), Nystatin 25 μg/mL and Progesterone 10 μg/mL (an inhibitor of caveolae-dependent pinocytosis).

### Detection of macropinocytosis by 3D-SIM imaging

Cells were incubated on 20 mm dishes. Different single antibody (40 nM) or combination of antibodies (20 nM each antibody) were treated on cells for 10 min, and the cell nuclei were dyed by Hoechst 33342. Then cells were imaged by DeltaVision OMX Imaging System, the imaging laser was 405 nm. When the imaging focus was moved to surface of cell membrane, the cup-like structure of macropinocytosis would be observed.

### Recombinant plasmid construction and expression of 7D12

The codons of wild-type 7D12 genes were optimized according to the PCR-based Accurate Synthesis, and the gene was cloned into pCznI vector. The recombinant 7D12 proteins were expressed in *E.coli* and the expression was induced by IPTG. 7D12 protein was purified by a nickel affinity column. The expression of 7D12 was detected by SDS-PAGE and western blotting.

### Plasmids and transfection

The plasmid pmApple and pmEmrald was kindly provided by Professor Pingyong Xu (Beijing, China). A probe consisting of the Pleckstrin homology (PH) domain and a fluorescent protein mApple (PIP2-PLCδ-mApple) used to label PIP2 in cell membrane. As inositol-polyphosphate 5-phosphatase Synaptojanin 2 (SJ2) can dephosphorylate the D5 position phosphate from PIP2, it could be used to consume PIP2 in cell membrane. Transfection of PH-PLCδ-mApple, FKBP_12_ and mEmrald-FRB-SJ2 was carried out using Lipofectamine 2000 according to the manufacturer’s instructions. Usually, cells were analyzed within 18–24 h following transfection.

### Statistical analysis

All assays were performed for at least three biological replicates. All statistical analyses were completed with GraphPad Prism 8 (GraphPad Software, San Diego, CA, USA). Data are presented as mean ± SD. All data were subjected to a nonparametric Kruskal–Wallis test to determine statistical significance.

## Results

### Imaging of lattice complex assembled by EGFR and its noncompetitive antibodies

**Fig. 1:**
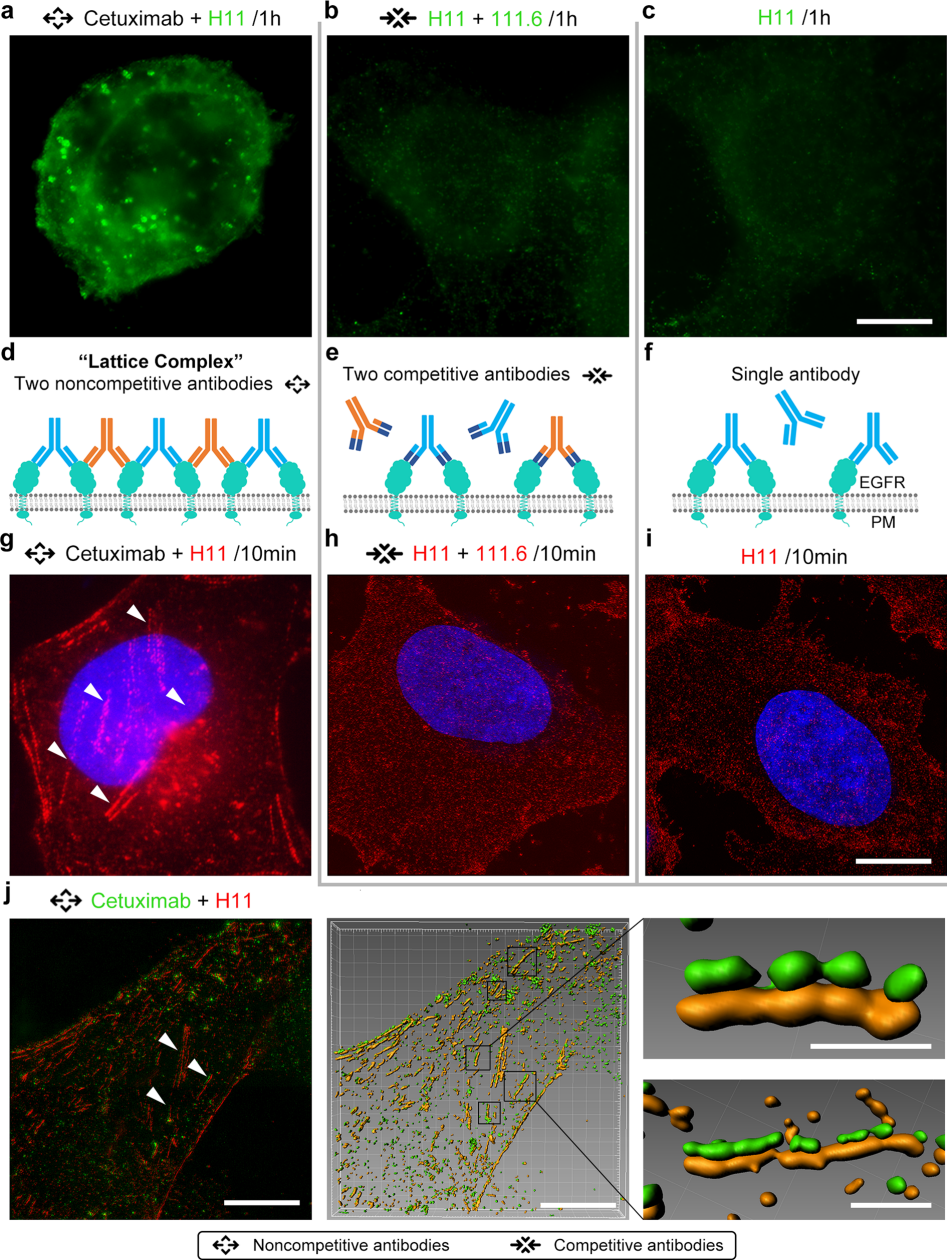
Lattice complex assembled by EGFR and its different antibody combinations on cell membrane. **a**–**c** Different single antibody (40 nM) or combination of antibodies (20 nM each antibody) was treated on Hela cells for 1 h. Bars, 10 μm. **d** The lattice complex formed by EGFR and its noncompetitive antibodies on cell membrane when two EGFR antibodies with noncompetitive epitopes were treated on Hela cells. **e** The EGFR pattern with two competitive antibodies on Hela cells. **f** EGFR-antibody complex formed on cell membrane by single antibody. **g, i** Different single antibody (40 nM) or combination of antibodies (20 nM each antibody) was treated on Hela cells for 10 min, cell nuclei were dyed by Hoechst 33342 (blue). Arrowheads showed the lattice complex. Bars, 10 μm. **j** 20 nM Cetuximab (green) and 20 nM H11 (red) were treated on Hela cells for 10 min. Arrowheads showed the lattice complex. Surface rendering was generated from Imaris software. Bars in left 2 columns, 10 μm; in right 1 column, 1 μm

In the previous studies, researchers outline the proposed model, accordingly, because of their bivalence, mAbs are able to form receptor homodimers, but treatment with combinations of mAbs will generate much larger receptor-Ab complexes. Stang et al. reported the imaging results that there was no distinct mAb mixture-induced clustering of HER2 at the plasma membrane [12]. Based on the different antibody combinations reported previously [11], Cetuximab + H11 and Cetuximab + 111.6 were used as the epitope noncompetitive antibody combination, and H11 + 111.6 was used as the competitive antibody combination (Additional file 1: Table S1). First, we compared internalization of EGFR on Hela cells treated with different antibody combinations for 1 h, noncompetitive antibody combinations increased the endocytosis in Hela cells (Fig. 1a–c, Additional file 2: Figure S1).

To investigate the behavior of antibodies on cell membrane, less treatment time (10 min) of antibodies was applied. Using a pair of noncompetitive EGFR antibodies (Cetuximab and H11, Cetuximab and 111.6, Fig. 1d) for lattice imaging on Hela cell surface, a large number of plaque-like, linear strips of red fluorescence were observed as the arrowheads shown in Fig. 1g. In contrast to these linear strips, with competitive EGFR antibodies (H11 and 111.6, Fig. 1e) and single antibody (H11, 111.6 or Cetuximab, Fig. 1f) stimulation, the antibodies (red fluorescence) were showed sporadically distributed uniformly on Hela cell surface (Fig. 1h, i, Additional file 3: Figure S2). The comparison of the above two results seems to prove the formation of the lattice complex on cell membrane with noncompetitive antibody combination. To further verify the existence of lattice complex, different fluorescent dyes were used to label Cetuximab (green) and H11 (red) respectively, and images were analyzed by Imaris Software. With the same experimental condition, similar lattice complex could be observed on cell membrane, and the distributed pattern of Cetuximab (green spots) in cells is very similar to that of H11 (red spots) (Fig. 1j). Similar results were observed in CaSki cells, another cervical cancer cell line (Additional file 4: Figure S3). These results further suggest that the two noncompetitive antibodies are able to form lattice complex on cell membrane.

### Symmetrical structure and different epitopes of antibody formed the lattice complex

**Fig. 2:**
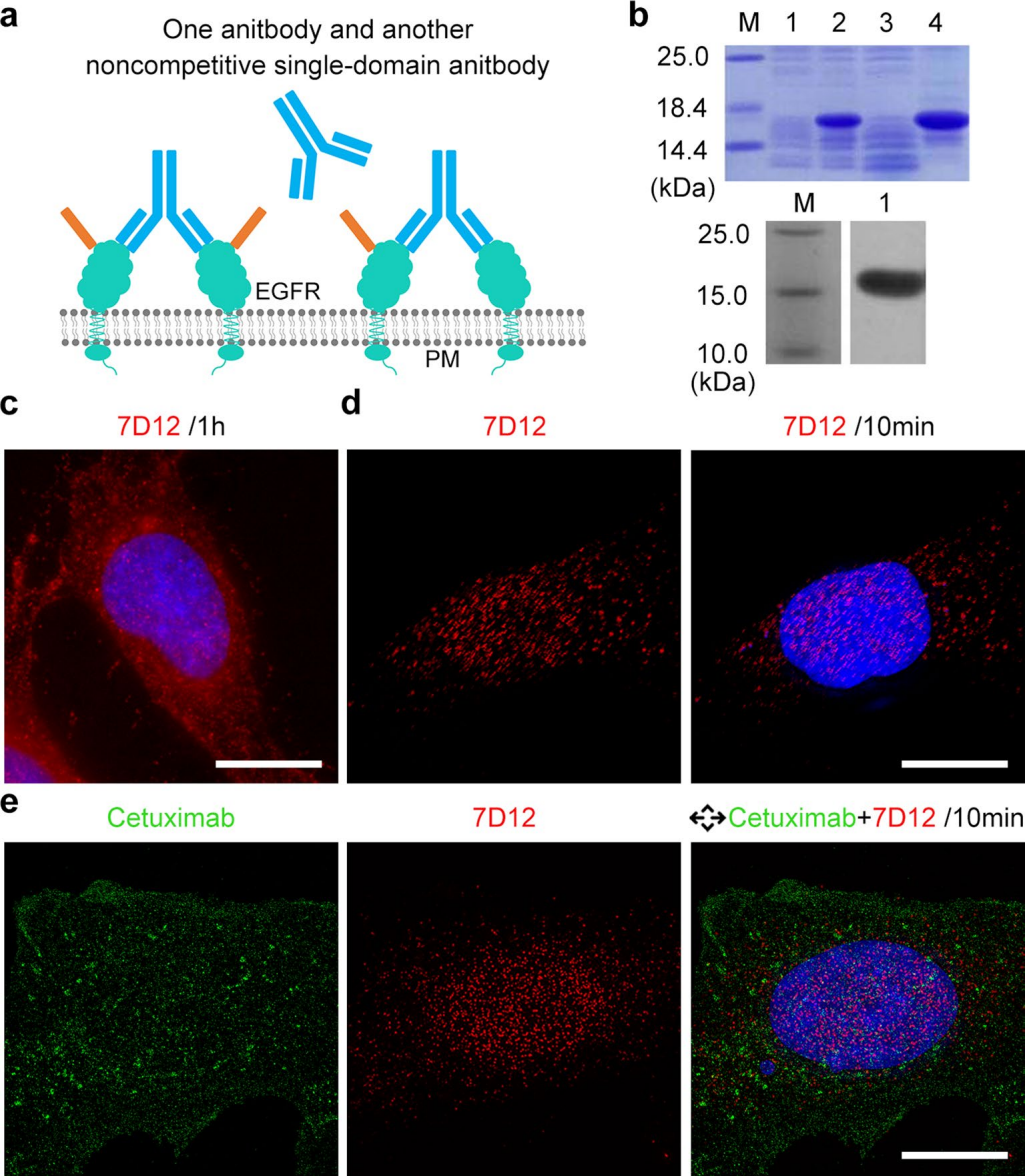
The expression and combination with its noncompetitive antibody of 7D12. **a** The EGFR-antibody complex with one antibody and another noncompetitive single domain antibody on Hela cells. **b** SDS-PAGE analysis of 7D12 after being carried out in *E. coli*. In SDS-PAGE imaging, lane M was protein marker, lane 1 was protein expression by non-induced *E. coli*, lane 2 was protein expression by induced *E. coli*, lane 3 was supernatant after *E. coli* disruption, lane 4 was sedimentation after *E. coli* disruption. In the western blotting analysis of 7D12 after purification, lane M was protein marker, lane 1 was protein after purification. **c** 40 nM 7D12 (red) was treated on Hela cells for 1 h, cell nuclei were dyed by Hoechst 33342 (blue). Bars, 10 μm. **d** 40 nM 7D12 (red) were treated on Hela cells for 10 min, cell nuclei were dyed by Hoechst 33342 (blue). Bars, 10 μm. **e** 20 nM Cetuximab (green) and 20 nM 7D12 (red) were treated on Hela cells for 10 min, cell nuclei were dyed by Hoechst 33342 (blue). Bars, 10 μm

Only the first part of the imaging results is still not enough to explain the existence of the lattice complex. Previous reports have speculated that the prerequisite for the formation of the lattice complex is the two symmetric Fab fragments of the antibody. To further verify this issue, another single-domain antibody (7D12) against EGFR, it possesses noncompetitive epitope with Cetuximab (Fig. 2a, Additional file 1: Table S1) [13]. 7D12 was overexpressed in *E.coli*, it was purified by a nickel affinity column NiTA-agarose and detected by SDS-PAGE and western blotting (Fig. 2b). As with other antibodies, 7D12 could enter Hela cells individually after 1 h treating (Fig. 2c).

When HeLa cells were treated with 7D12 and Cetuximab (Fig. 2d, e), no linear lattice complex was formed on cell membrane, in contrast to our observations with the noncompetitive Cetuximab + H11. Consistent with other individual antibody, Fig. 2d, e showed similar uniform tiny spots after 7D12 treatment alone. This in turn suggests that the observed linear lattice complex was formed by bridging EGFR molecules recognized by the symmetrical Fab fragments of two noncompetitive antibodies.

### Noncompetitive antibody combination triggered high-intensity internalization by macropinocytosis

**Fig. 3:**
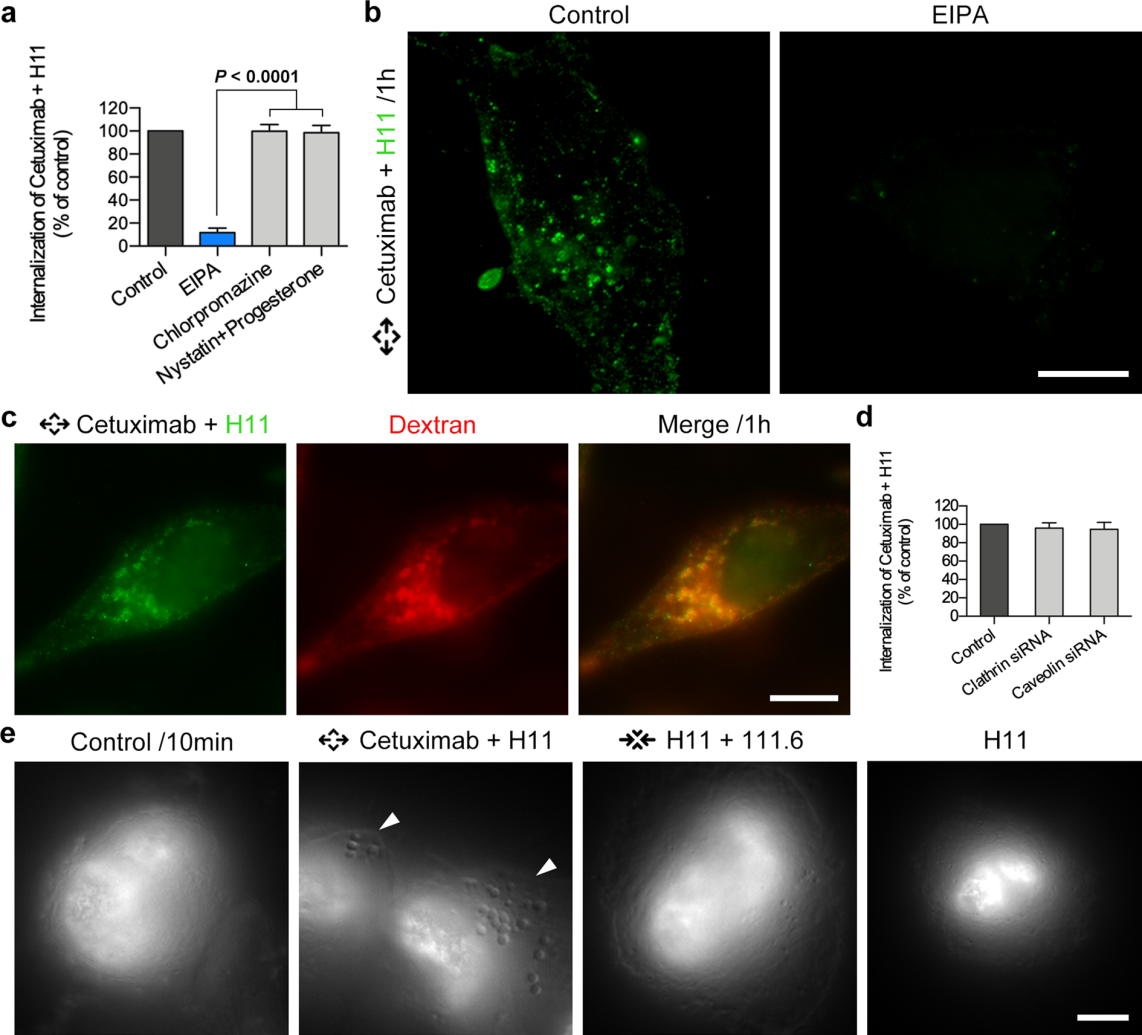
Noncompetitive antibody combination triggered high intensity internalization by macropinocytosis. **a**, **b** 20 nM Cetuximab and 20 nM H11 (green) were treated on Hela cells for 1 h after different inhibitors pre-incubated for 30 min. All data are mean ± SD. Bars, 10 μm. Statistical significance was calculated with a nonparametric Kruskal–Wallis test, in which a *P *< 0.0001 was considered significant. **c** 20 nM Cetuximab and 20 nM H11 (green) and 100 μg/mL Dextran or BSA (red) co-treated on Hela cells for 1 h before cells imaging. Bars, 10 μm. **d** 20 nM Cetuximab and 20 nM H11 were treated on Hela cells for 1 h after clathrin/caveolin was interfered by its siRNA. All data are mean ± SD. **e** Hela cell nuclei were dyed by Hoechst 33342 after different single antibody (40 nM) or combination of antibodies (20 nM each antibody) treating for 10 min. Arrowheads showed the cup-shaped macropinosomes. Bars, 5 μm

After the existence of the lattice complex was confirmed, we further explored the relationship between the lattice complex and high intensity internalization caused by combination of noncompetitive antibodies. To clarify how lattice complex enter the cells, we used the inhibitors for clathrin-dependent pinocytosis, caveolae-dependent pinocytosis and macropinocytosis, respectively. EIPA, an inhibitor of macropinocytosis, inhibited internalization of Cetuximab + H11 significantly compared with other inhibitors (Fig. 3a, b). As shown in Fig. 3c, the marker of macropinocytosis (dextran) was co-localized with noncompetitive antibodies distinctly after 1 h incubation in Hela cells. Besides, the internalization did not change significantly after the expression of clathrin and caveolin being inhibited (Fig. 3d). To further confirm the endocytosis pathway, we used an established macropinocytosis detection method to investigate endocytosis with noncompetitive antibodies [14]. Cup-shaped macropinosomes were observed to assemble on cell membrane with noncompetitive antibody combination, and similar results were not observed with single antibody or competitive antibody combination treating (Fig. 3e, Additional file 5: Figure S4). Each cup exhibited a similar diameter (0.7–0.9 μm), with cups assembling like linear strips. Taken together, noncompetitive antibody combination may cause high intensity macropinocytosis in Hela cells.

### The lattice complex assembles along F-actin on cell membrane

**Fig. 4:**
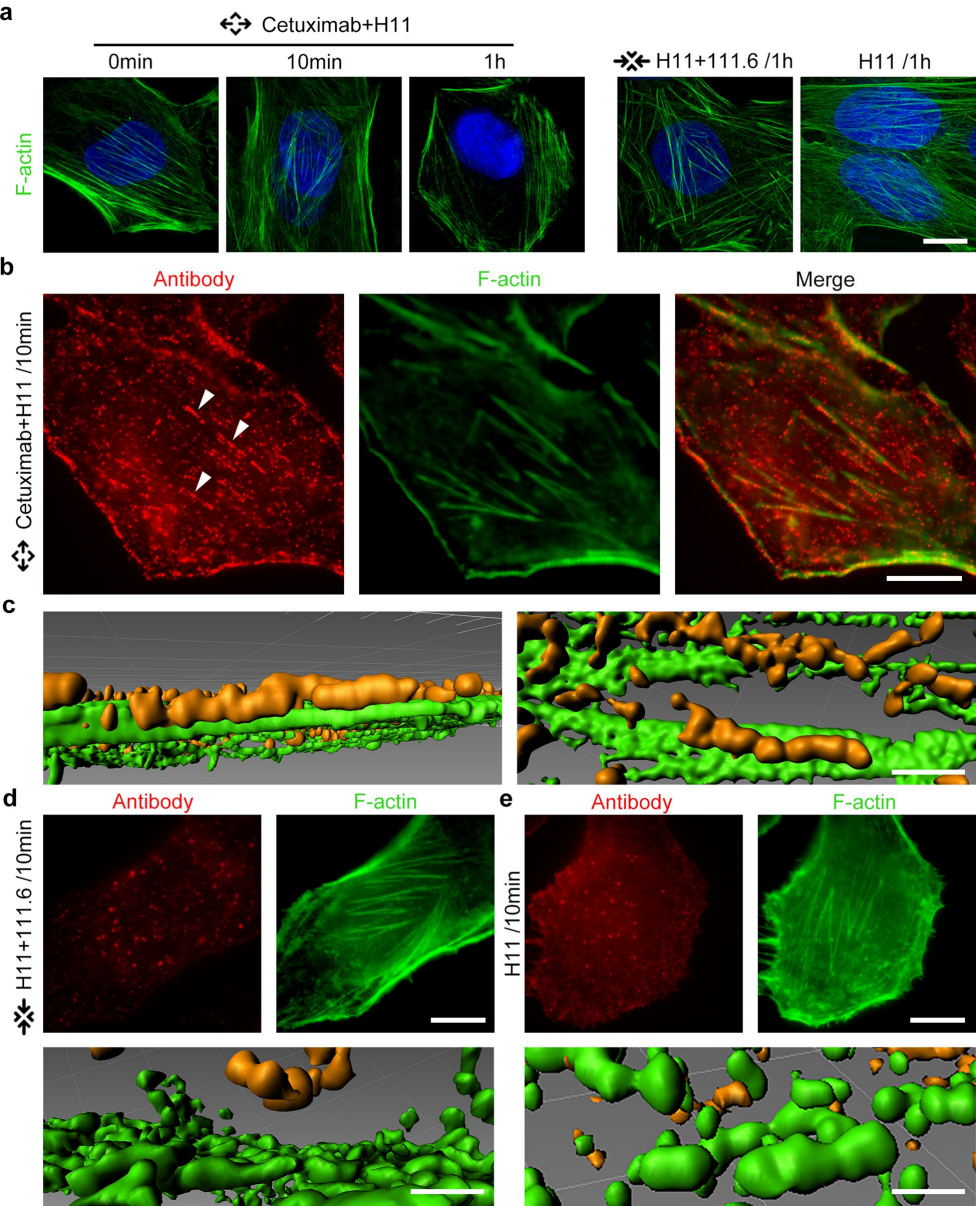
Lattice complex assembled along F-actin on cell membrane. **a** Different single antibody (40 nM) or combination of antibodies (20 nM each antibody) was treated on Hela cells for 10 min or 1 h, cell nuclei were dyed by Hoechst 33342 (blue). Afterward, F-actin was stained with AlexaFluor 488 Phalloidin. Bars, 10 μm. **b** 20 nM Cetuximab and 20 nM H11 (red) were treated on Hela cells for 10 min, F-actin was stained with AlexaFluor 488 Phalloidin. Arrowheads showed the lattice complex. Bars, 10 μm. **c** Surface rendering of images from (**b**) was generated from Imaris software. Bars, 1 μm. **d**, **e** Different combination of antibodies (20 nM each antibody) or single antibody (40 nM) was treated on Hela cells for 10 min, F-actin was stained with AlexaFluor 488 Phalloidin. Surface rendering of images was generated from Imaris software. Bars above, 10 μm, bars below, 10 μm

Above large size lattice complex may be closely related to high intensity internalization of antibody. As the reorganization of F-actin is a major feature of macropinocytosis, we observed the pattern variation of F-actin with antibody combination. After noncompetitive antibody combinations were treated on Hela cells for 1 h, F-actin on the ventral cell surface position reorganized and could not be observed. There was no changes of F-actin under competitive antibody or single antibody combination (Fig. 4a). Considering previous results, the micron linearity of lattice complex on cell membrane is particularly striking and it seems to align in a nonrandom pattern (Fig. 1g).

To investigate the changes in F-actin during the lattice complex internalization, we focus on the positional relationship of the distribution of two noncompetitive antibodies and F-actin in the initial stage of the lattice complex assembly. We treated Hela cells by Cetuximab and H11 as noncompetitive antibody combination for 10 min. Antibodies (red) and F-actin (green) were labeled by different fluorescent dyes. Surprisingly, the F-actin and antibodies were located on similar positions (Fig. 4b). With further analysis of this distribution, the arrangement of these two linear fluorescent spots on cell membrane exhibited significantly same pattern, while red and green fluorescence showed clear co-localization (Fig. 4c), which seemed that the lattice complex was distributed as F-actin on cell membrane. Moreover, in the H11 + 111.6 and individual antibody group, the similar distributed pattern was not been observed (Fig. 4d, e, Additional file 6: Figure S5). Similar distribution could be also observed in CaSki cells (Additional file 7: Figure S6). Overall, these results indicate that lattice complex formed by noncompetitive antibody combination runs along cytoskeletal fibers on cell membrane. The most striking result to emerge from the data is that the arrangement of lattice complex with F-actin may cause the high-intensity internalization of noncompetitive antibodies. This makes us wonder that the large-size lattice complex on the cell surface affects the cytoskeleton and membrane transport cycle, which may also be the main reason for its internalization.

### Noncompetitive antibody combination induces a PIP2 consumption dependent internalization

The mechanism of lattice formation causing high intensity internalization in cells is interesting. From the pattern of lattice complex, there are more EGFR and relative molecules of internalization accumulating on cell membrane after noncompetitive antibody combinations, and macropinocytosis involves recruitment and fusion of cell membrane regions, as well as actin polymerization. And the lattice complex runs along F-actin on cell membrane. These bring us to focus on phosphatidylinositol-4,5-bisphosphate (PIP2). PIP2 plays a crucial role in this process [15, 16]. PIP2 is also critical for EGFR cluster formation and activity [17]. Thus, we investigated the roles of PIP2 in the formation of lattice and high intensity macropinocytosis.

**Fig. 5:**
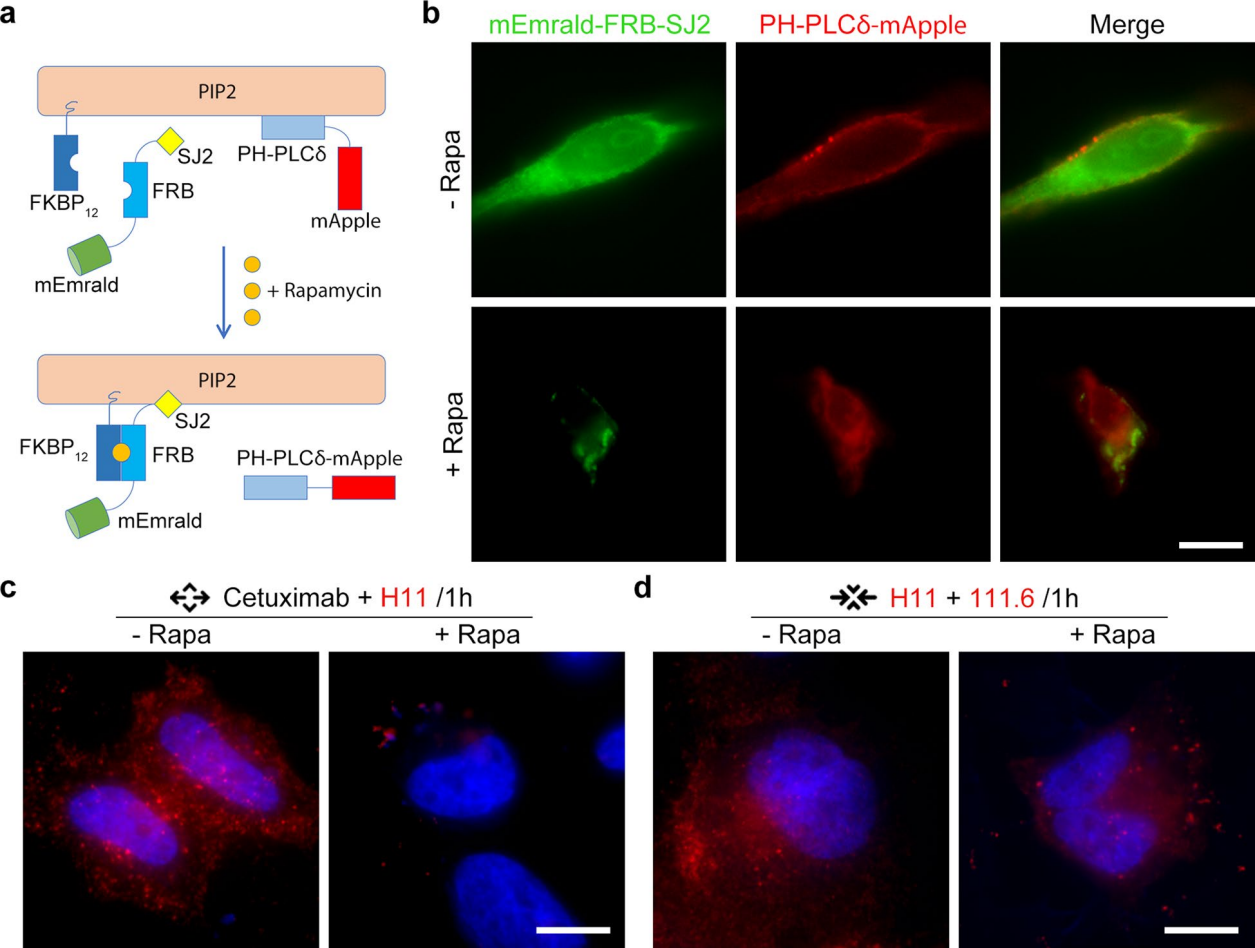
The establishment of PIP2 consumption system and its impairment on noncompetitive antibody combination. **a** Schematics of the establishment of PIP2 consumption system. **b** Validation of the PIP2 consumption system in Hela cells. Bars, 10 μm. **c**, **d** Different combination of antibodies (20 nM each antibody) were treated for 1 h on Hela cells transfected with the PIP2 consumption system before (−Rapa) and after (+Rapa) rapamycin treatment. Bars, 10 μm

Based on a previous report [18], a PIP2 consumption system was established (Fig. 5a). A probe (PIP2-PLCδ-mApple) was used to label PIP2 in cell membrane. To control the process of PIP2 consumption, we constructed two plasmids: FKBP_12_ and mEmrald-FRB-SJ2. FKBP_12_ localizes in plasma membrane under normal condition, while mEmrald-FRB-SJ2 is targeted to cytoplasm. The treatment of Rapamycin could trigger the heterodimerization of FRB and FKBP12 domains, thus SJ2 is targeted to plasma membrane for PIP2 consumption (Fig. 5a). Our data showed that the position of red and green fluorescence exchanged in Hela cells after Rapamycin treatment (Fig. 5b).

Then the effect of PIP2 consumption on internalization of combinative antibodies was studied. With three plasmids (FKBP_12_, mEmrald-FRB-SJ2 and PH-PLCδ-mApple) transfecting to Hela cells, the internalization of Cetuximab + H11 was observed. After Rapamycin treatment, there was almost no red fluorescence in cytoplasm, the internalization was significantly inhibited (Fig. 5c). But the similar inhibition was not occurred on internalization of H11 + 111.6 (Fig. 5d). In CaSki cells, PIP2 consumption could also interfer the internalization of noncompetitive antibody combination (Additional file 8: Figure S7). Besides, the formation of lattice complex was not interfered (data not shown). The above results indicate that noncompetitive antibodies in combination mediated transport across cell membrane and F-actin polymerization, then increased endocytosis of EGFR by high intensity macropinocytosis. Interestingly, the large-size lattice complex seemed to affect membrane fluidity and dynamic reorganization of cytoskeletal, which may be responsible for its rapid internalization.

## Discussion

Due to complex intracellular pathways and autologous abnormal activation of EGFR, the marketed EGFR-targeted drugs are often confronted with drug resistance or failure in clinical trials [19–21]. Thus, it is essential to develop the new target or mechanism of EGFR targeted therapy. In 2017, Sym004, a synergistic antibody combination containing two antibodies which bind to different epitopes of EGFR, was been proven to provide survival benefit or stabilization for patients with mCRC that have acquired resistance to Cetuximab [8, 9]. And it has been reported that Sym004 could induce rapid internalization and degradation of EGFR [5]. It seems that the key point of the anti-tumor mechanism is abundant EGFR internalization causing by noncompetitive antibody combination.

Thus researchers proposed a lattice complex formed by EGFR and its noncompetitive antibodies which may related to these intense internalization [11]. Evidences of the lattice complex by now are obtained mostly by western blotting or flow cytometry, there is no direct evidence available in favor of the lattice complex on cell membrane. By 3D-SIM imaging technique, we observed micron linear fluorescent spots on cell membrane after noncompetitive antibody combination, whereas not occurring with competitive antibodies. Meanwhile, as the relationship between antibody structure and lattice formation, we used a single-domain antibody with only one variable region in heavy chain, combined with its noncompetitive antibody, there is no similar lattice formation on cell membrane. Above all, these results provide direct evidences for the existence of lattice complex. Besides, the symmetrical structure and different epitopes of antibody are the key factors to form the lattice complex.

It is significant to explore the relationship between this lattice complex and high intensity internalization. One interesting finding is the nonrandom pattern of lattice complex on cell membrane, and we found that lattice complex arranged along the cytoskeletal fibers. Since the reorganization of F-actin was a major feature of macropinocytosis and the occur of macropinocytosis needed abundant F-actin, with noncompetitive antibody combination, the antibodies and EGFR assembled along F-actin and recruited relative molecules to induce the rearrangement of F-actin and subsequent macropinosome formation. Taken these data together with the inhibition of EGFR internalization caused by the consumption of PIP2, a crucial regulator in F-actin polymerization, these results suggest that the lattice complex assembled by noncompetitive anti-EGFR antibodies could trigger a F-actin relevant EGFR internalization. Thus noncompetitive antibody combination could induce rapid internalization and latter inhibition of cell growth. More molecular mechanism of EGFR in lattice complex still need to be revealed in further studies.

Cytoskeleton and cell membrane occurred self-recycle continuously, and PIP2 could regulate F-actin reorganized and cell membrane transported on the process of endocytosis [22, 23]. The whole system constituted the stability and dynamic balance of cells. According to our results, the lattice complex formed assembling along F-actin and then caused reorganization of F-actin, it seemed to affect the stability and balance of cytoskeleton. Some inhibitors of cytoskeleton, such as trichostatin A [24, 25], have been reported to be anti-tumor drugs, the combination of noncompetitive antibodies and trichostatin A may get better benefit in the clinic, and similar combination could be further explored and applied.

In summary, this study provides direct evidences on the formation of lattice complex on cell membrane induced by noncompetitive antibody combination. It also preliminarily demonstrated that high intensity macropinocytosis caused by lattice complex is F-actin relevant. These findings shed new light on the selection and therapeutic regimen of antibody combinations in the clinic. They also provide optimizing ways for antibody structure, location of design and development of new anti-EGFR agents.

## Conclusions

The most significant finding to emerge from this study is that two antibodies with noncompetitive epitopes could conjugate EGFR on cell membrane to form a large lattice complex, the lattice complex assembled along with cytoskeletal fibers, its internalization recruited a large amount of PIP2 and triggered the rearrangement of F-Actin. In general, it seems that large-size lattice complex affects membrane fluidity and dynamic reorganization of cytoskeletal, which may be responsible for its rapid internalization.

## Supplementary information

**Additional file 1: Table S1.** Binding sites of anti-EGFR antibodies.

**Additional file 2: Figure S1.** Internalization of EGFR on Hela cells treated with different antibody combinations in Hela cells.

**Additional file 3: Figure S2.** Lattice complex assembled by EGFR and its different antibody combinations on cell membrane in Hela cells.

**Additional file 4: Figure S3.** Intrnalization and lattice complex assembled by EGFR and its different antibody combinations in CaSki cells.

**Additional file 5: Figure S4.** Cup-shaped macropinosomes on cell membrane with different antibody combinations.

**Additional file 6: Figure S5.** Lattice complex assembled along F-actin on cell membrane in Hela cells.

**Additional file 7: Figure S6.** Lattice complex assembled along F-actin on cell membrane in CaSki cells.

**Additional file 8: Figure S7.** PIP2 consumption could interfere the internalization of noncompetitive antibody combination in CaSki cells.

## Data Availability

The datasets used and analyzed during the current study are available from the corresponding author on reasonable request.

## Abbreviations

EGFR: Epitomal growth factor receptor
EIPA: 5-(N-ethyl-N-isopropyl)-Amiloride
IPTG: Isopropyl β-D-1-thiogalactopyranoside
mAb: Monoclonal antibody
mCRC: Metastatic colorectal cancer
PBS: Phosphate-buffered saline
PCR: Polymerase chain reaction
PH: Pleckstrin homology
PIP2: Phosphatidylinositol-4,5-bisphosphate
SJ2: Synaptojanin 2
3D-SIM: Three-dimensional structured illumination microscopy
