# Supplementary material for: Lattice complex assembled by noncompetitive anti-EGFR antibodies regulates actin cytoskeletal reorganization
Source: Cancer Cell Int. 2020 Apr 21;20:129. doi: 10.1186/s12935-020-01204-z (PMC7171787; doi:10.1186/s12935-020-01204-z)
Supplement: Supplementary file 1 — Additional file 1: Table S1. Binding sites of anti-EGFR antibodies. [file 12935_2020_1204_MOESM1_ESM.docx]

**Table S1** **Binding sites of anti-EGFR antibodies**

| Antibody | Binding sites | EGFR extracellular domain |
| --- | --- | --- |
| Cetuximab | Q384, Q408, H409, K443, K465, I467, N473 | Domain III |
| H11 | S356, H359 | Domain III |
| 111.6 | R353, S356, H359 | Domain III |
| 7D12 | R353, D355, F357 | Domain III |
