# Supplementary figures and images for: Lattice complex assembled by noncompetitive anti-EGFR antibodies regulates actin cytoskeletal reorganization
Source: Cancer Cell Int. 2020 Apr 21;20:129. doi: 10.1186/s12935-020-01204-z (PMC7171787; doi:10.1186/s12935-020-01204-z)

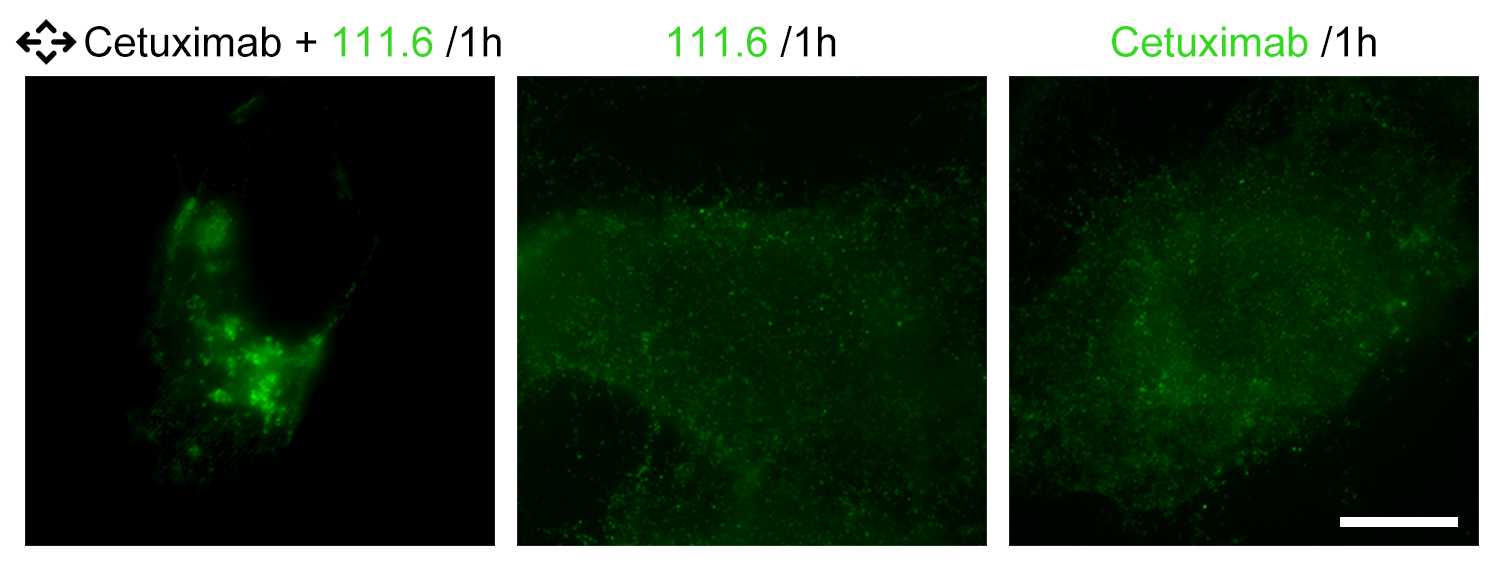

Supplement: Supplementary file 2 — Additional file 2: Figure S1. Internalization of EGFR on Hela cells treated with different antibody combinations in Hela cells. [file 12935_2020_1204_MOESM2_ESM.tif]

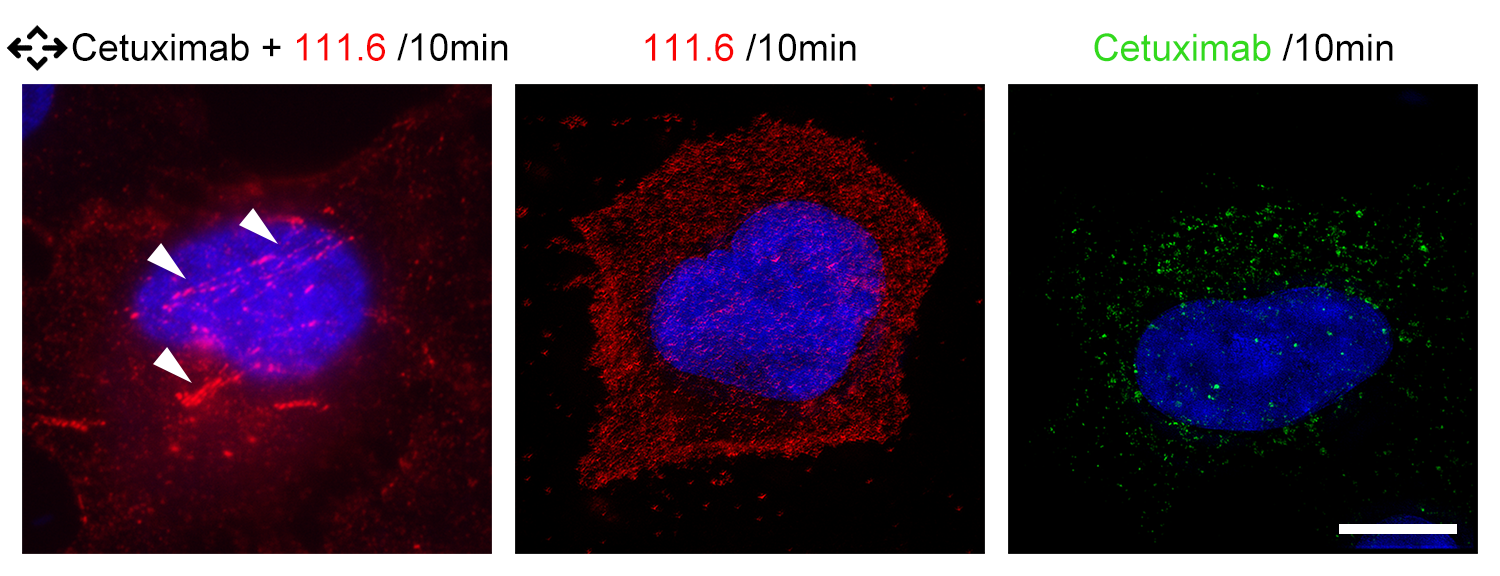

Supplement: Supplementary file 3 — Additional file 3: Figure S2. Lattice complex assembled by EGFR and its different antibody combinations on cell membrane in Hela cells. [file 12935_2020_1204_MOESM3_ESM.tif]

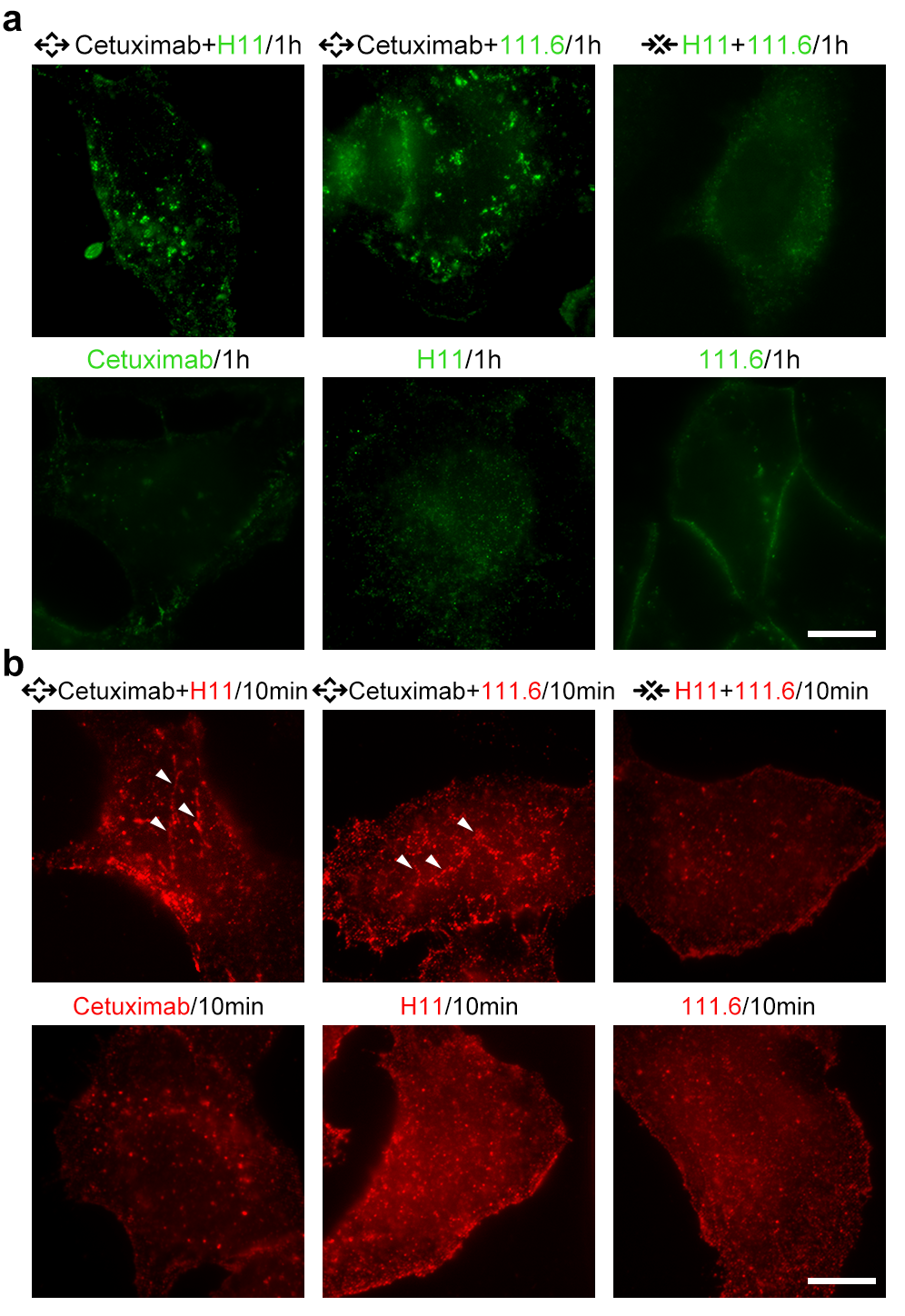

Supplement: Supplementary file 4 — Additional file 4: Figure S3. Intrnalization and lattice complex assembled by EGFR and its different antibody combinations in CaSki cells. [file 12935_2020_1204_MOESM4_ESM.tif]

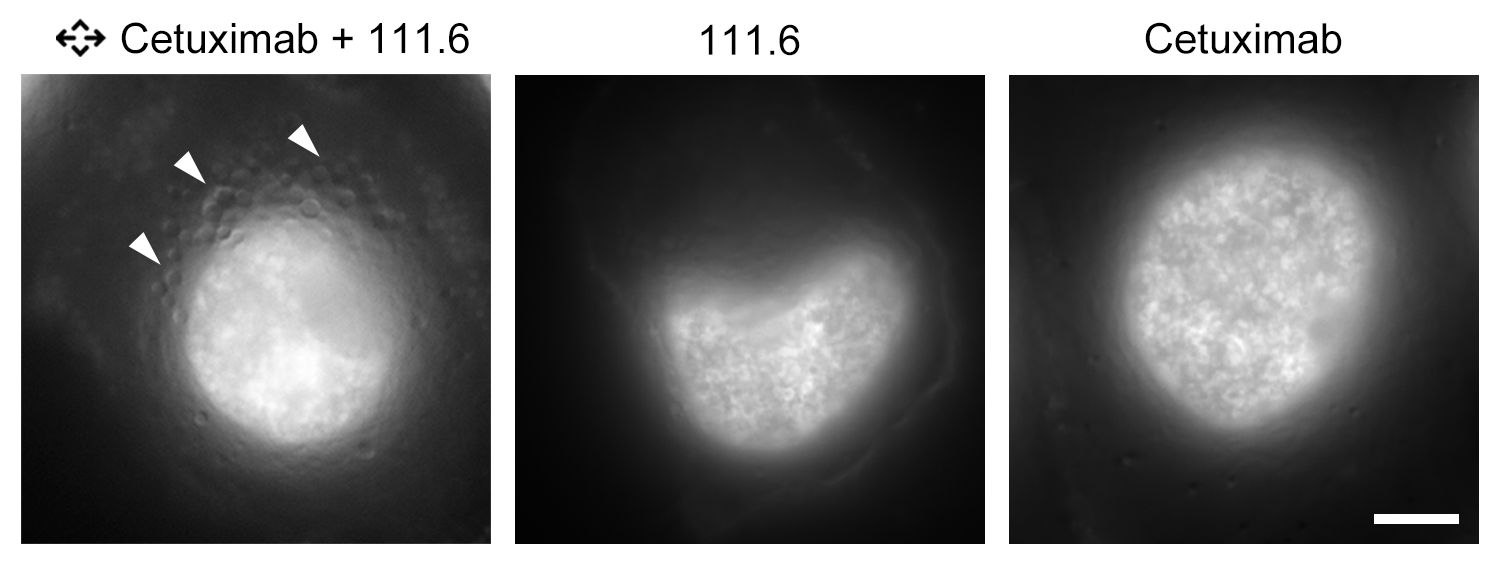

Supplement: Supplementary file 5 — Additional file 5: Figure S4. Cup-shaped macropinosomes on cell membrane with different antibody combinations. [file 12935_2020_1204_MOESM5_ESM.tif]

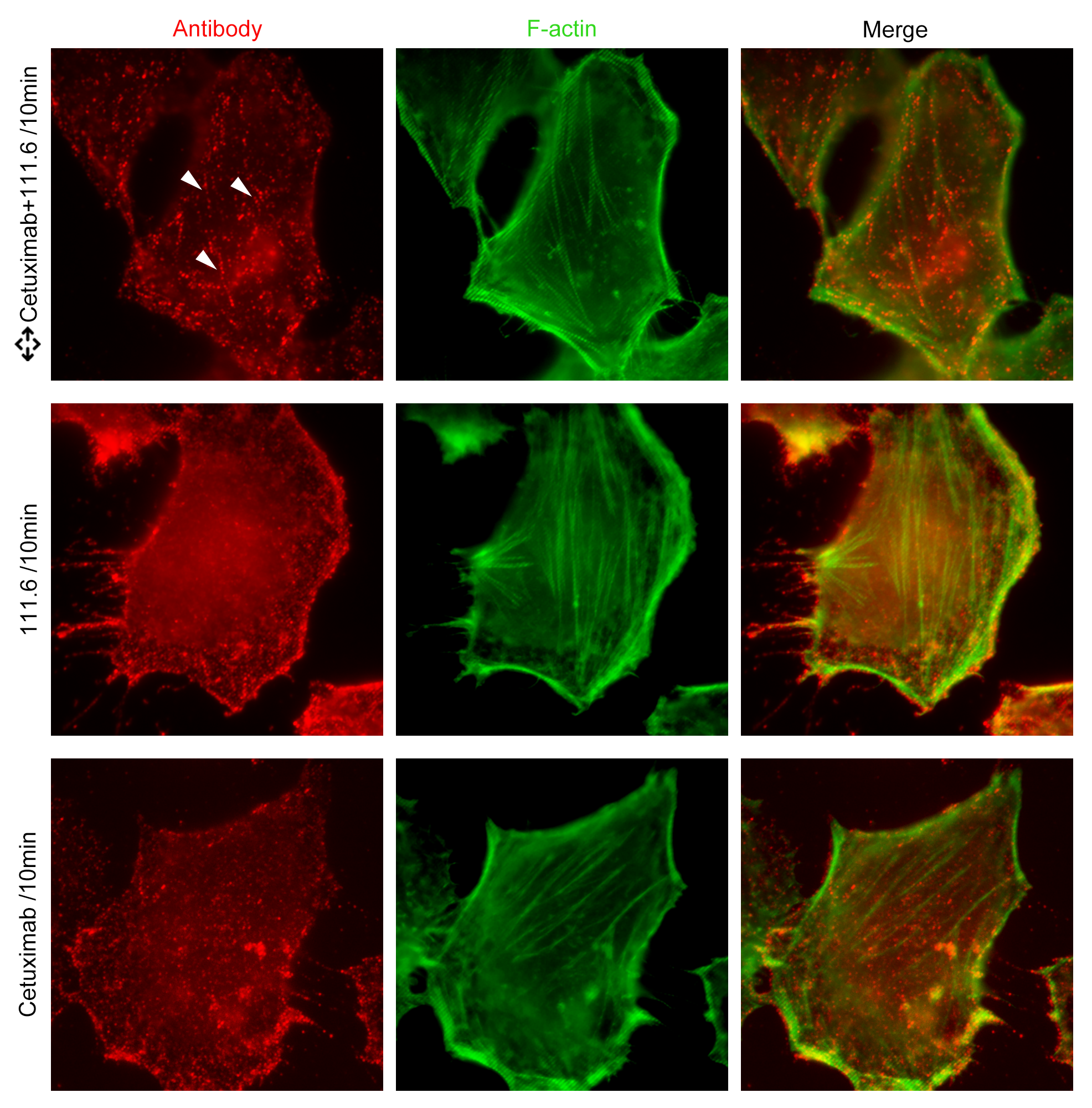

Supplement: Supplementary file 6 — Additional file 6: Figure S5. Lattice complex assembled along F-actin on cell membrane in Hela cells. [file 12935_2020_1204_MOESM6_ESM.tif]

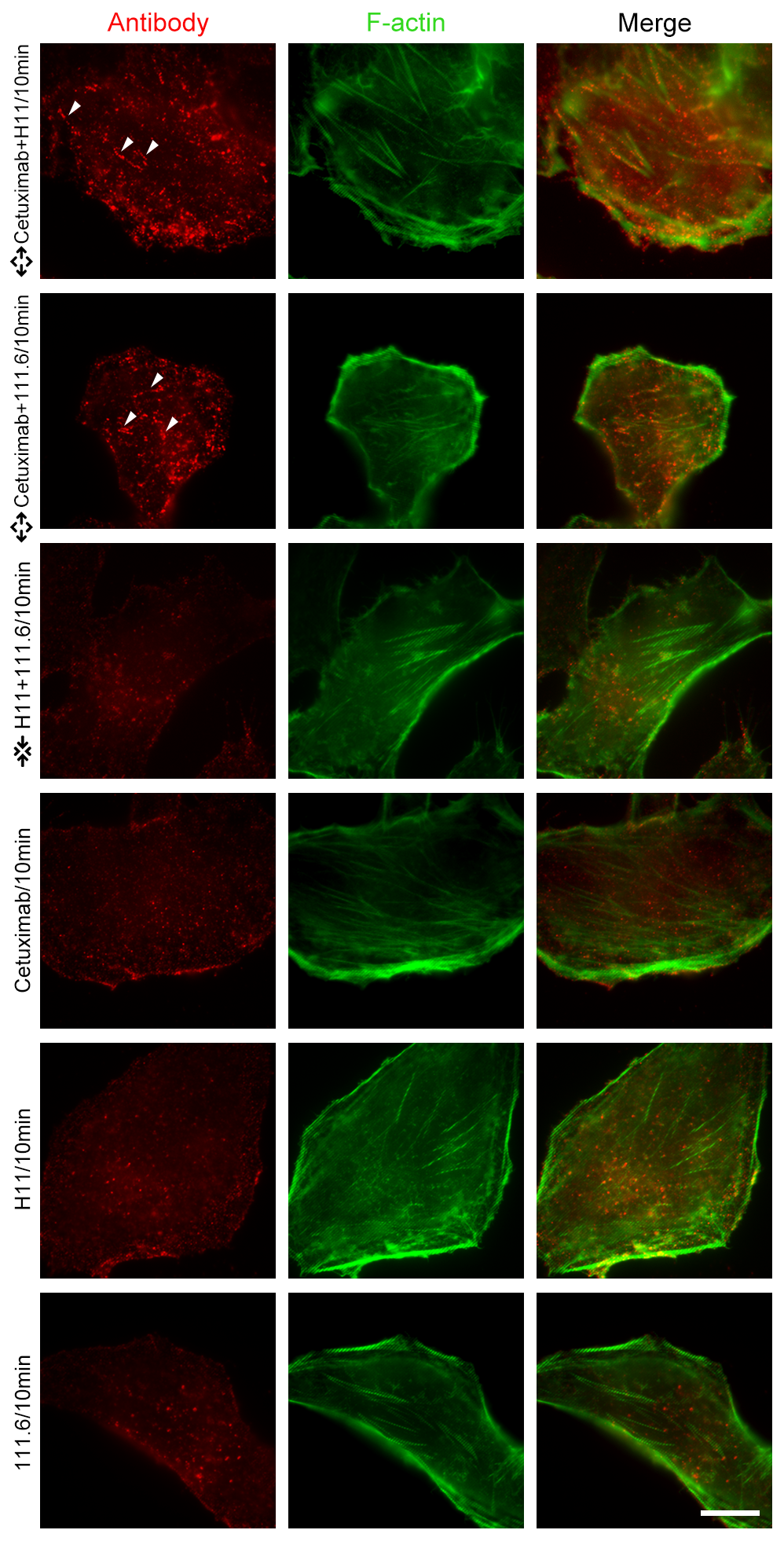

Supplement: Supplementary file 7 — Additional file 7: Figure S6. Lattice complex assembled along F-actin on cell membrane in CaSki cells. [file 12935_2020_1204_MOESM7_ESM.tif]

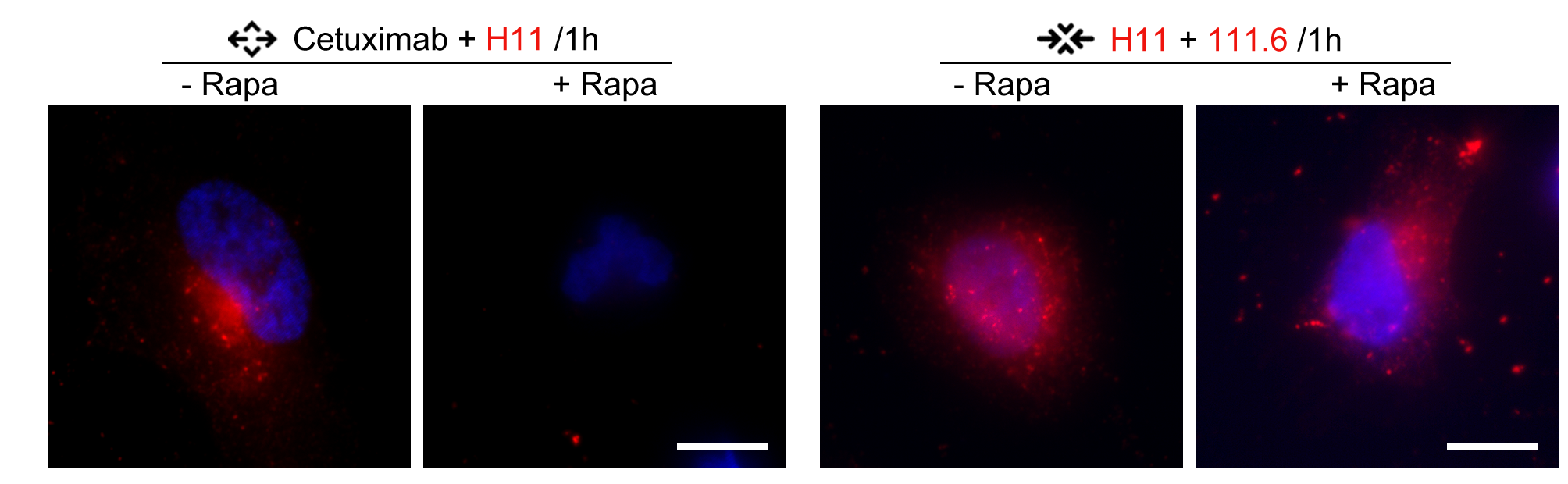

Supplement: Supplementary file 8 — Additional file 8: Figure S7. PIP2 consumption could interfere the internalization of noncompetitive antibody combination in CaSki cells. [file 12935_2020_1204_MOESM8_ESM.tif]
